# Voluntary intake of psychoactive substances is regulated by the dopamine receptor Dop1R1 in *Drosophila*

**DOI:** 10.1038/s41598-021-82813-0

**Published:** 2021-02-09

**Authors:** Mai Kanno, Shun Hiramatsu, Shu Kondo, Hiromu Tanimoto, Toshiharu Ichinose

**Affiliations:** 1 Graduate School of Life Sciences, Tohoku University, Sendai, 980-8577 Japan; 2 Invertebrate Genetics Laboratory, National Institute of Genetics, Mishima, 411-8540 Japan; 3 Frontier Research Institute for Interdisciplinary Sciences, Tohoku University, Sendai, 980-8578 Japan; 4 Center for Transdisciplinary Research, Niigata University, Niigata, 950-2181 Japan; 5 Department of Neuropharmacology, Nagoya City University, Nagoya, 467-8603 Japan

**Keywords:** Reward, Genetics of the nervous system, Addiction

## Abstract

Dysregulated motivation to consume psychoactive substances leads to addictive behaviors that often result in serious health consequences. Understanding the neuronal mechanisms that drive drug consumption is crucial for developing new therapeutic strategies. The fruit fly *Drosophila melanogaster* offers a unique opportunity to approach this problem with a battery of sophisticated neurogenetic tools available, but how they consume these drugs remains largely unknown. Here, we examined drug self-administration behavior of *Drosophila* and the underlying neuronal mechanisms. We measured the preference of flies for five different psychoactive substances using a two-choice feeding assay and monitored its long-term changes. We found that flies show acute preference for ethanol and methamphetamine, but not for cocaine, caffeine or morphine. Repeated intake of ethanol, but not methamphetamine, increased over time. Preference for methamphetamine and the long-term escalation of ethanol preference required the dopamine receptor Dop1R1 in the mushroom body. The protein level of Dop1R1 increased after repeated intake of ethanol, but not methamphetamine, which correlates with the acquired preference. Genetic overexpression of Dop1R1 enhanced ethanol preference. These results reveal a striking diversity of response to individual drugs in the fly and the role of dopamine signaling and its plastic changes in controlling voluntary intake of drugs.

## Introduction

Substance use disorders cause severe human health problems, including more than 350 thousand direct and many more indirect deaths per year^1^. Contribution of genetic factors to the risk of drug addiction is estimated to be around half^2^, highlighting the importance of animal models that enable investigation of the genetic underpinnings.

The fruit fly *Drosophila melanogaster* has been established as a useful genetic model organism for decades. These flies show many characteristic responses common in humans, especially toward ethanol. Ethanol is known to target several ion channels, protein kinase C and adenylate cyclase^3^. Exposure of the flies to vapored ethanol make them first hyperactive, uncoordinated, and eventually sedated^4–6^. They develop tolerance after repeated exposure^7,8^ and show withdrawal-like symptoms when ethanol is suddenly withheld after chronic exposure^9^. These flies indeed like ethanol: food odor attraction is enhanced by the addition of ethanol^10,11^, and they prefer to feed or lay eggs on food containing ethanol^12,13^. When an odor cue coincides with ethanol exposure, they form associative reward memory^14,15^. Importantly, self-administration of ethanol-containing food increases after several days of drinking or acute exposure to the drug^12,16–18^, capturing one of the most critical steps toward the formation of alcohol use disorder^19,20^.

Effects of other drugs have also been examined in *Drosophila*. X-ray crystallography showed that methamphetamine and cocaine bind to the *Drosophila* dopamine transporter^21^, which is the primary molecular target of these drugs in mammals^22^. Similar to humans, methamphetamine and cocaine prevent sleep and cause hyperactivity in a dopamine-dependent manner^23–25^. Caffeine also causes insomnia through dopamine and the cAMP pathway^26–29^. Opioid, which targets several G-protein coupled receptors known as opioid receptors in mammals, affects development and lifespan of flies^30–32^. An opiate antagonist is reported to suppress alcohol preference in flies^17^, implying functional conservation. Because these drugs often cause compulsive drug seeking behavior in humans, it is tempting to hypothesize that flies may also choose to consume these drugs.

Previous studies indicated the relevance of the reward circuit in some of these behaviors. Natural reward, such as sugar or water, is conveyed by a cluster of dopamine neurons called protocerebral anterior medial (PAM) to the mushroom body (MB)^33–37^. Prolonged exposure of ethanol vapor activates PAM cluster neurons^38^, and blocking PAM neurons or the MB intrinsic or output neurons impairs the ethanol-rewarded olfactory memory^14,38–40^. PAM and the MB intrinsic neurons also influences ethanol preference for oviposition^13^. Self-administration of ethanol is regulated by neuropeptide-F^41^, which also mediates reward^42^, and a cytoskeletal regulator in the MB^43^. However, involvement of dopamine and the MB circuit for ethanol drinking remains to be elucidated.

In this study, we aimed to obtain a systematic overview of voluntary intake of five different drugs (ethanol, cocaine, caffeine, morphine and methamphetamine) using two-choice capillary feeding (CAFE) assay^44^. We found that flies prefer to drink ethanol and methamphetamine, but not cocaine, caffeine and morphine. Moreover, dopamine-Dop1R1 signaling in the mushroom body (MB) and its experience-dependent change mediate ethanol and methamphetamine preference. These results reveal diverse responses of flies toward different drugs and the underlying neuronal mechanisms.

## Results

### Systematic investigation of drug feeding behavior of flies

**Figure 1:**
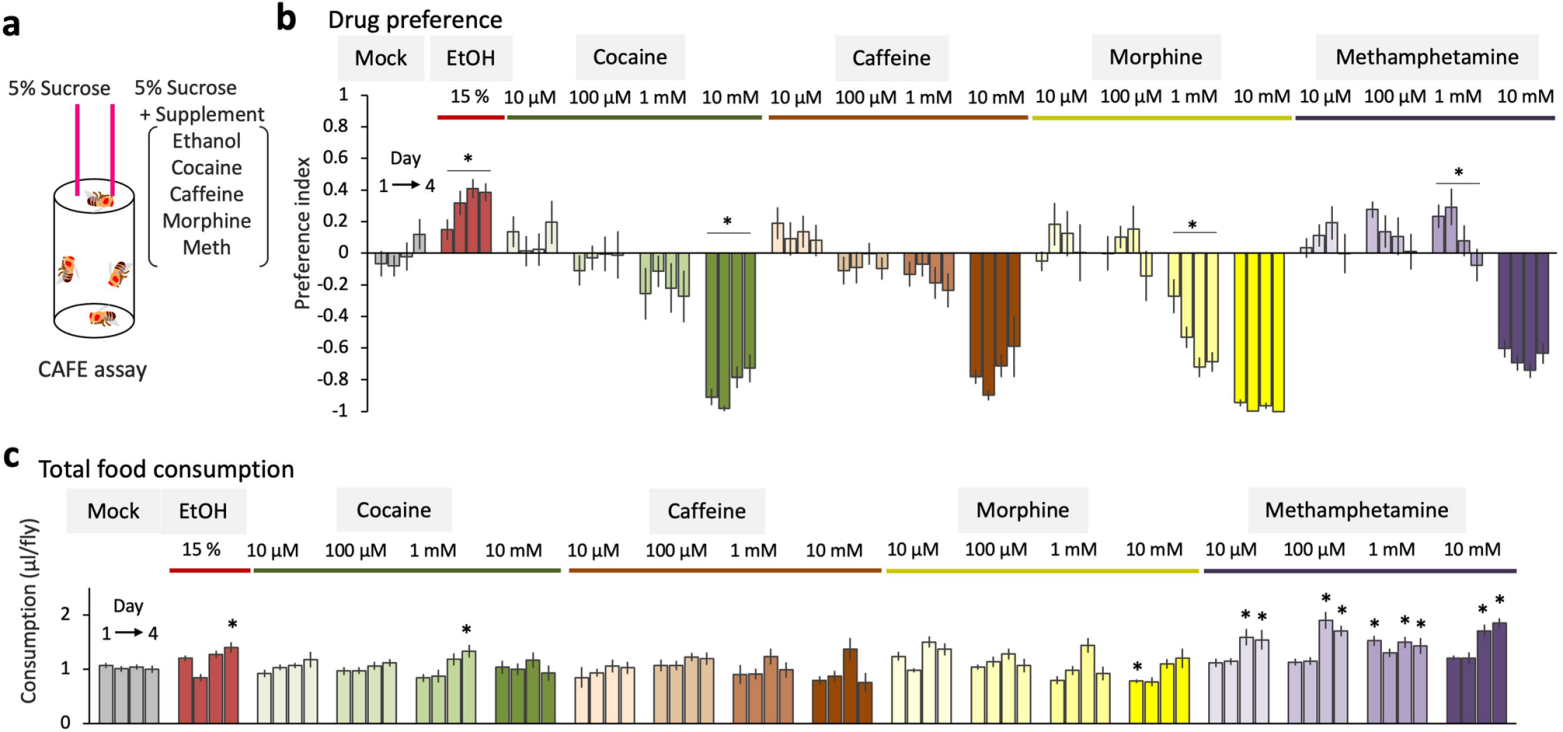
The *Drosophila* drug intake behavior. (**a**) Schematics of the two-choice CAFE assay. A group of four male flies is given a choice between 5% (w/v) sucrose solution and 5% sucrose solution supplemented with drugs. Daily consumption of each solution is measured as a descent of the meniscus for 4 days. (**b**) Preference indices of the wild type male flies are plotted from the day 1 to the day 4. Concentration of the drug is indicated above the bar graphs. One-way repeated-measures ANOVA or the Friedman test is performed among the indices from the day 1 to 4. 15% EtOH: *F*_(2.654, 53.08)_ = 6.743, *P* = 0.0010, *n* = 21 (ANOVA); 10 µM cocaine: *P* = 0.3013, *n* = 11 (Friedman); 100 µM cocaine: *F*_(1.610, 16.10)_ = 0.4848, *P* = 0.5845, *n* = 11 (ANOVA); 1 mM cocaine: *F*_(1.433, 15.76)_ = 0.4714, *P* = 0.5700, *n* = 12 (ANOVA); 10 mM cocaine: *P* = 0.0088, *n* = 8 (Friedman); 10 µM caffeine: *F*_(2.482, 27.30)_ = 0.4237, *P* = 0.7012, *n* = 12 (ANOVA); 100 µM caffeine: *F*_(2.047, 18.42)_ = 0.7174, *P* = 0.5042, *n* = 10 (ANOVA); 1 mM caffeine: *P* = 0.2881, *n* = 11 (Friedman); 10 mM caffeine: *P* = 0.1904, *n* = 10 (Friedman); 10 µM morphine: *F*_(1.880, 15.04)_ = 1.546, *P* = 0.2448, *n* = 9 (ANOVA); 100 µM morphine: *F*_(2.025, 20.25)_ = 2.003, *P* = 0.1604, *n* = 11 (ANOVA); 1 mM morphine: *P* = 0.0011, *n* = 11 (Friedman); 10 mM morphine: *F*_(1.522, 15.22)_ = 3.251, *P* = 0.0773, (ANOVA); 10 µM methamphetamine: *F*_(2.100, 29.40)_ = 1.166, *P* = 0.3277, *n* = 15 (ANOVA); 100 µM methamphetamine: *F*_(2.695, 43.12)_ = 1.816, *P* = 0.1634, *n* = 17 (ANOVA); 1 mM methamphetamine: *F*_(2.450, 41.64)_ = 4.581, *P* = 0.0111, *n* = 18 (ANOVA); 10 mM methamphetamine: *P* = 0.2572, *n* = 15 (Friedman). Bar graphs: mean ± SEM. *: *P* < 0.05. (**c**) Daily food consumption per fly. Total consumption of the control and the drug solution is plotted. Consumption of each group is compared to that of the mock group on the same day (Two-stage linear step-up procedure of Benjamini, Krieger and Yekutieli). 15% EtOH: *q* = 0.3330, 0.7398, 0.1127, 0.0031 (from the day 1 to 4); 10 µM cocaine: *q* = 0.3827, 0.9714, 0.8151, 0.1205; 100 µM cocaine: *q* = 0.6909, 0.8851, 0.8151, 0.1985; 1 mM cocaine: *q* = 0.2488, 0.8851, 0.3234, 0.0210; 10 mM cocaine: *q* = 0.8374, 0.9714, 0.4366, 0.5297; 10 µM caffeine: *q* = 0.8281, 0.9714, 0.3234, 0.2297; 100 µM caffeine: *q* = 0.3330, 0.6240, 0.0891, 0.1020; 1 mM caffeine: *q* = 0.8281, 0.8851, 0.2266, 0.0736; 10 mM caffeine: *q* = 0.2488, 0.8566, 0.0891, 0.6174; 10 µM morphine: *q* = 0.9166, 0.8851, 0.2495, 0.1985; 100 µM morphine: *q* = 0.6909, > 0.9999, 0.4768, 0.5297; 1 mM morphine: *q* = 0.0607, 0.8305, 0.3608, 0.5463; 10 mM morphine: *q* = 0.0295, 0.6240, 0.8151, 0.3114; 10 µM methamphetamine: *q* = 0.8281, 0.7398, 0.0099, 0.0051; 100 µM methamphetamine: *q* = 0.0609, 0.8244, 0.0002, 0.0001; 1 mM methamphetamine: *q* = 0.0295, 0.3329, 0.0099, 0.0068; 10 mM methamphetamine: *q* = 0.3330, 0.6240, 0.0003, 0.0001. Bar graphs: mean ± SEM. *: *P* < 0.05. Bar graphs: mean ± SEM. *: *P* < 0.05.

To measure the drug preference and its experience-dependent changes, we performed two-choice CAFE assay^44^. A group of four wild type male flies was given a choice between 5% sucrose solution and 5% sucrose solution supplemented with five different drugs in different concentrations (ethanol, cocaine, caffeine, morphine or methamphetamine) (Fig. 1a). Daily consumption of each solution was measured for 4 days, and the drug preference index was calculated (“Methods”). Consistent with the previous report^12^, flies showed a mild preference for ethanol on the first day, which escalated on the following days (Fig. 1b). In contrast, they showed a dose-dependent aversion to caffeine, cocaine and morphine throughout the measurement (Fig. 1b). Aversion to morphine developed over time, suggesting a negative reinforcing effect of the drug (Fig. 1b). Interestingly, methamphetamine was preferred on the first day at 100 µM or 1 mM, but the preference gradually diminished on the following days (Fig. 1b). Ethanol and methamphetamine increased the total food consumption—the sum of consumed sucrose solutions irrespective of drug presence (Fig. 1c), which might be because of the hyperactivity induced by these drugs^23,25,45^. Altogether, these results revealed distinct dynamics of drug intake behavior of flies.

### Repeated drug feeding underlies the preference

**Figure 2:**
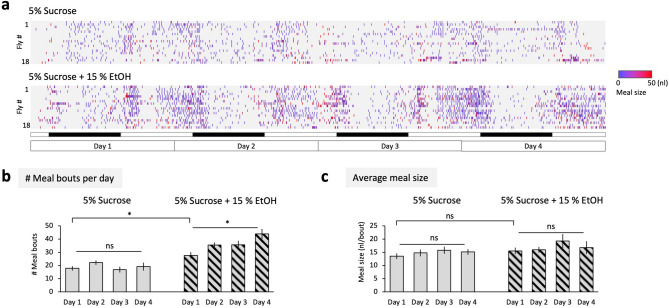
Escalation of repeated intake of ethanol. (**a**) Raster plots of meal-bouts of 5% sucrose solution (upper) and 5% sucrose supplemented with 15% ethanol (lower). Flies are offered with both solution similar to Fig. 1. Note that flies are measured individually not to mix up the consumption of multiple flies. Level of meniscus is measured every 5 minutes and the drop bigger than mean + 2*SD evaporation is defined as a meal bout. Rows and columns respectively indicate individual flies and time bins, and the open and filled open boxes below indicate light and dark cycle (12–12 h). Size of each meal is shown with color as shown on the right. (**b**) The number of detected meal bouts per day. 5% sucrose: *F*_(2.018, 34.30)_ = 1.408, *P* = 0.2585, *n* = 18 (one-way repeated-measures ANOVA); 5% sucrose + 15% EtOH: *F*_(1.669, 28.38)_ = 7.710, *P* = 0.0034, *n* = 18 (one-way repeated-measures ANOVA). 5% sucrose vs 5% sucrose + 15% EtOH on the day 1: *t* = 2.698, *P* = 0.0152, *n* = 18 (paired *t*-test). (**c**) Average meal size. 5% sucrose: *F*_(2.452, 41.68)_ = 0.9641, *P* = 0.4047, *n* = 18 (one-way repeated-measures ANOVA). 5% sucrose + 15% EtOH: *P* = 0.0719, *n* = 18 (Friedman test). 5% sucrose vs 5% sucrose + 15% EtOH on the day 1: *P* = 0.1187, *n* = 18 (Wilcoxon test). Bar graphs: mean ± SEM. *: *P* < 0.05.

**Figure 3:**
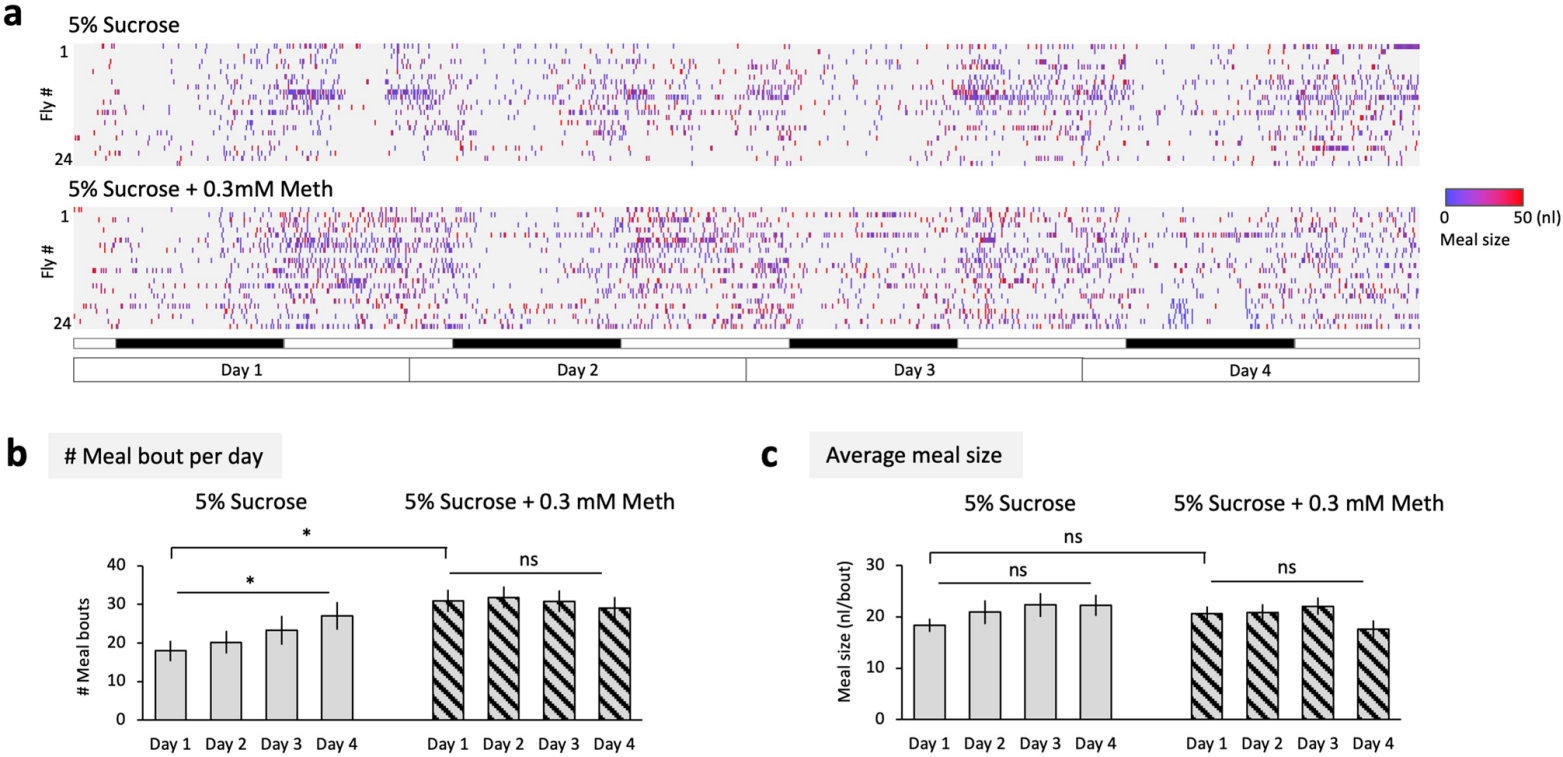
Repeated intake of methamphetamine. (**a**) Raster plots of meal-bouts of 5% sucrose solution (upper) and 5% sucrose supplemented with 0.3 mM methamphetamine (lower). This dose is chosen because both 0.1 and 1 mM methamphetamine elicit the drug preference (see Fig. 1b). Meal events and the size are measured and shown in a similar manner to the Fig. 2. (**b**) The number of detected meal bouts per day. 5% sucrose: *P* = 0.0273, *n* = 24 (Friedman test); 5% sucrose + 0.3 mM methamphetamine: *P* = 0.7756, *n* = 24 (Friedman test). 5% sucrose vs 5% sucrose + 0.3 mM methamphetamine on day1: *P* = 0.0047, *n* = 24 (Wilcoxon test). (**c**) Average meal size. 5% sucrose: *P* = 0.1337, *n* = 24 (Friedman test); 5% sucrose + 0.3 mM methamphetamine; *F*_(1.648, 37.89)_ = 2.547, *P* = 0.1008, *n* = 24 (one-way repeated-measures ANOVA). 5% sucrose vs 5% sucrose + 0.3 mM methamphetamine on the day 1: *P* = 0.2076, *n* = 24 (Wilcoxon test). Bar graphs: mean ± SEM. *: *P* < 0.05.

One of the hallmarks of drug addiction is repeated drug intake. We therefore next asked if the preference for ethanol or methamphetamine comes from more frequent feeding on drug-containing food or increased meal size on it. To dissociate these two possibilities, we estimated the number of feeding events and the average meal size of a single-housed fly by measuring the liquid level every 5 minutes (Figs. 2a and 3a). Interestingly, we found that the flies drank ethanol- or methamphetamine-containing solution more often than the control on the first day (Figs. 2b and 3b). The average size of each meal on the other hand was not significantly different (Figs. 2c and 3c). The frequency of ethanol intake, but not the average meal size, progressively increased over time (Fig. 2b,c). Conversely when methamphetamine was provided, flies became to consume the control sucrose solution more often while frequency of methamphetamine feeding stayed rather constant across the 4 days (Fig. 3b). Therefore, we conclude that the drug preference is shaped by repeated drug feeding, but not by different size of each meal.

### Dop1R1 signaling in the mushroom body regulates drug preference

**Figure 4:**
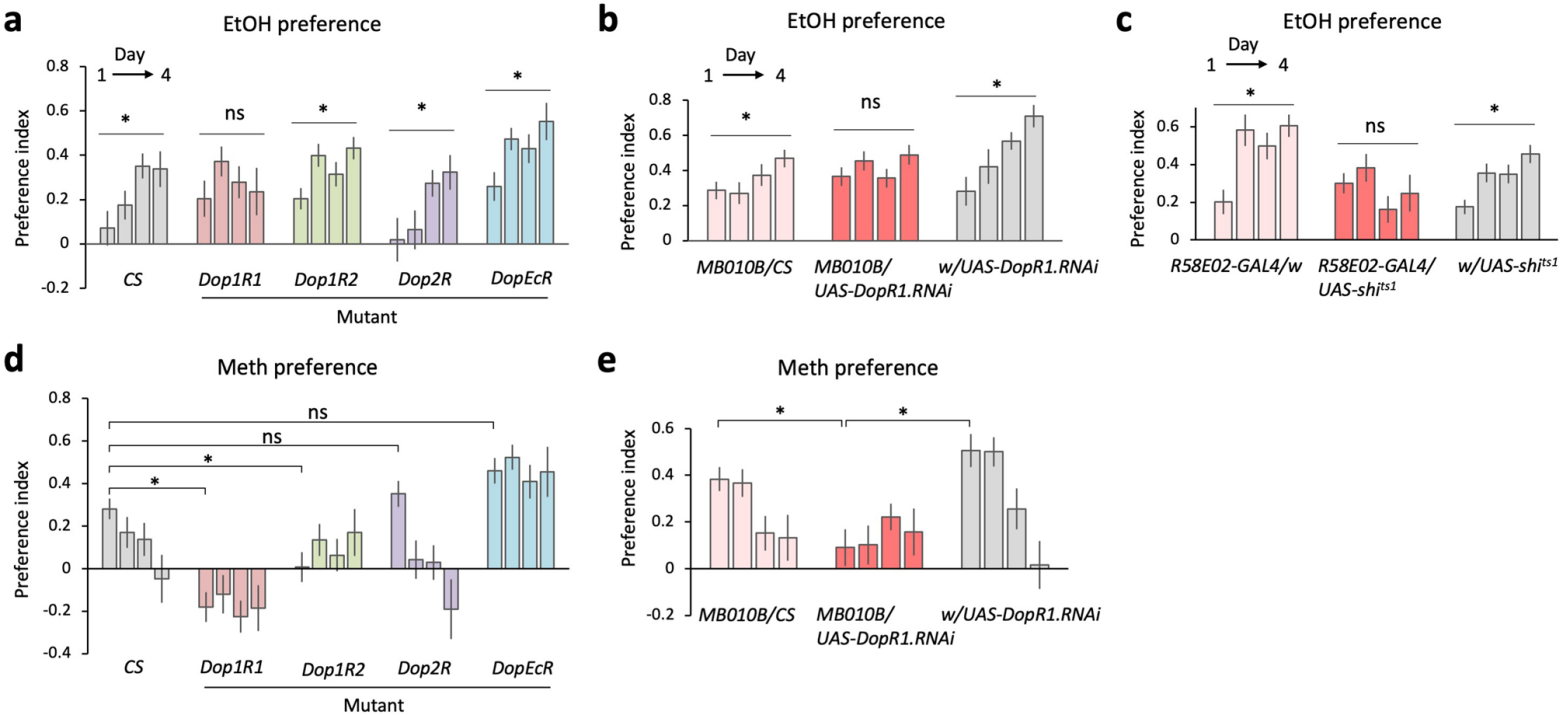
Dop1R1 signaling in the mushroom body mediates ethanol and methamphetamine preference. (**a**) *Dop1R1* is necessary for acquired ethanol preference. Daily preference for 15% ethanol is plotted for the wild type (*CS*) and mutant strains for the four dopamine receptors. One-way repeated-measures ANOVA or the Friedman test is performed among the indices from the day 1 to 4. *CS*: *P* < 0.0001, *n* = 24 (Friedman); *Dop1R1*: *P* = 0.2004, *n* = 22, (Friedman); *Dop1R2*: *F*_(2.176, 43.52)_ = 6.715, *P* = 0.0023, *n* = 21 (ANOVA); *Dop2R*: *F*_(1.676, 38.55)_ = 5.037, *P* = 0.0155, *n* = 24 (ANOVA); *DopEcR*: *F*_(2.021, 40.42)_ = 5.731, *P* = 0.0063, *n* = 21 (ANOVA). (**b**) *Dop1R1* expression in the mushroom body is necessary for acquired ethanol preference. *Dop1R1* is genetically knocked down using the *MB010B-GAL4* driver. *MB010B/CS*: *F*_(1.719, 56.73)_ = 4.093, *P* = 0.0271, *n* = 34 (ANOVA); *MB010B/UAS-Dop1R1.RNAi*: *F*_(2.806, 78.57)_ = 2.282, *P* = 0.0896, *n* = 29 (ANOVA); *w/UAS-Dop1R1.RNAi*: *F*_(1.979, 43.54)_ = 6.227, *P* = 0.0043, *n* = 23 (ANOVA). (**c**) Neurotransmission from the PAM cluster dopamine neurons is necessary for acquired ethanol preference. PAM cluster neurons are blocked throughout the measurement using the *R58E02-GAL4* and the *UAS-shibire*^*ts1*^ strains and the 15% ethanol preference is plotted. *R58E02-GAL4/w*: *F*_(2.663, 47.93)_ = 9.746, *P* < 0.0001, *n* = 19 (ANOVA); *R58E02-GAL4/UAS-shi*^*ts1*^: *F*_(2.382, 52.41)_ = 1.718, *P* = 0.1840, *n* = 23 (ANOVA); *w/UAS-shi*^*ts1*^: *F*_(2.705, 119.0)_ = 6.435, *P* = 0.0007, *n* = 45 (ANOVA). Note that female flies were used for the measurement because of the high mortality when males were used (see also Supplementary Figure 1). (**d**) *Dop1R1* is necessary for acute methamphetamine preference. Daily preference for 0.3 mM methamphetamine is plotted for the wild type (*CS*) and mutant strains for the four dopamine receptors. The drug preference of mutant strains is compared to that of the wild type on the first day (Dunnett’s multiple comparison). *CS* vs *Dop1R1*: *P* < 0.0001, *CS* vs *Dop1R2*: *P* = 0.0052, *CS* vs *Dop2R*: *P* = 0.8207, *CS* vs *DopEcR*: *P* = 0.2005. (**e**) *Dop1R1* expression in the mushroom body is necessary for acute methamphetamine preference. *Dop1R1* is genetically knocked down using the *MB010B-GAL4* driver. The drug preference on the first day is compared among genotypes (Dunn’s multiple comparison). *MB010B-GAL4/CS* vs. *MB10B-GAL4/UAS-DopR1.RNAi*: *P* = 0.0193; *MB010B-GAL4/UAS-DopR1/RNAi* vs. *w/UAS-DopR1.RNAi*: *P* = 0.0001. Bar graphs: mean ± SEM. *: *P* < 0.05.

Ethanol and methamphetamine induce dopamine release in the fly brain^21,38,45–47^. Therefore, we tested the role of dopamine receptors in the preference for ethanol and methamphetamine by using mutants of the four dopamine receptors: *Dop1R1*, *Dop1R2*, *Dop2R* and *DopEcR*. We found that the mutant of *Dop1R1*, but not of the other receptors, failed to acquire the experience-dependent ethanol preference (Fig. 4a). The *Dop1R1* mutant however showed a normal or slightly enhanced ethanol preference on the first day (Fig. 4a), suggesting that the acute ethanol preference is *Dop1R1*-independent. In contrast, the same mutation suppressed the preference for methamphetamine throughout the measurement (Fig. 4d). *Dop1R2* mutation also suppressed the methamphetamine preference (Fig. 4d). In contrast, the *DopEcR* mutant showed continuously high preference for both drugs (Fig. 4a,d), suggesting its role in the drug aversion. Because sugar and ethanol reward is conveyed to the mushroom body (MB) in the context of associative learning^33,38,48,49^, we next tested the role of Dop1R1 in the MB. Genetic knock-down of *Dop1R1* in the MB showed a consistent phenotype to the mutant, highlighting the importance of the MB in this behavior (Fig. 4b,e). Consistently, blockade of the reward-conveying PAM cluster dopamine neurons using shibire^ts1^^50^ abolished the acquired ethanol preference (Fig. 4c). Altogether, these results highlight the importance of the Dop1R1 signaling in the MB for the drug preference but in a distinct manner: acquired preference for ethanol and basal preference for methamphetamine.

**Figure 5:**
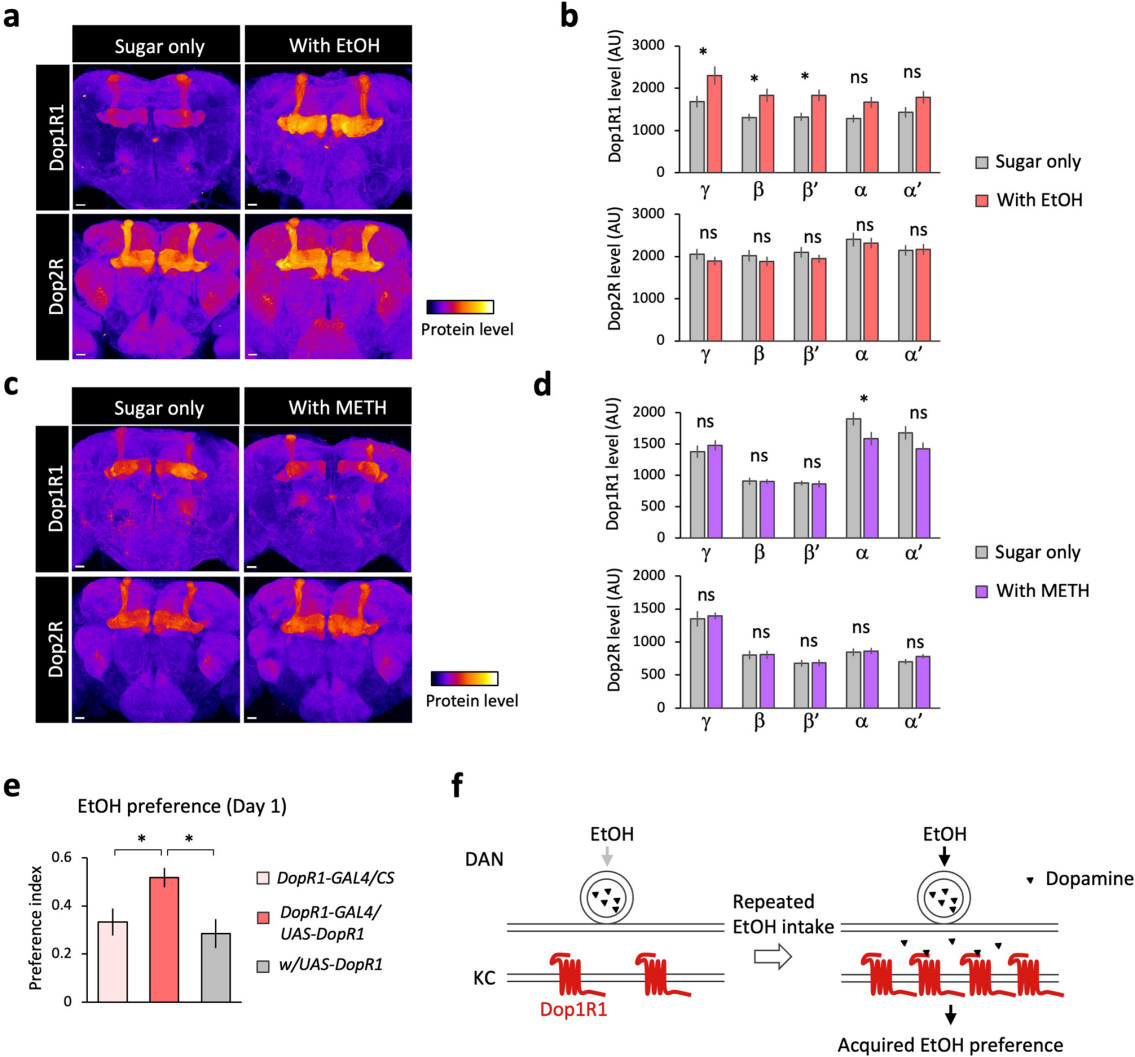
Experience-dependent increase of Dop1R1 protein mediates acquired preference for ethanol. (**a**) Ethanol intake increases the protein level of Dop1R1-Venus, but not Dop2R-Venus. Representative images of the z-projection are shown. Flies are offered with only 5% sucrose solution (left, “Sugar only”) or a choice of 5% sucrose and 5% sucrose with 15% ethanol (right, “With EtOH”) for 3 days in the CAFE assay. (**b**) Increased Dop1R1 (up) or Dop2R (down) protein in the medial lobe of the MB. Mean fluorescent intensity of Dop1R1- or Dop2R-Venus at the slice of the mushroom body lobes is compared between the groups (Sidak’s multiple comparison). Dop1R1; γ lobe: *P* = 0.0075, β lobe: *P* = 0.0350, β′ lobe: *P* = 0.0405, α lobe: *P* = 0.2044, α′ lobe: *P* = 0.2809. *n* = 16 and 14 for “Sugar only” and “With EtOH”, respectively. Dop2R; γ lobe: *P* = 0.9008, β lobe: *P* = 0.9467, β′ lobe: *P* = 0.9151, α lobe: *P* = 0.9928, α′ lobe: *P* > 0.9999. *n* = 8 and 11 for “Sugar only” and “With EtOH”, respectively. (**c**) Methamphetamine does not increase the protein level of Dop1R1 or Dop2R. Flies were offered with only 5% sucrose solution or a choice of 5% sucrose and 5% sucrose with 0.3 mM methamphetamine for 3 days in the CAFE assay. (**d**) Quantification of level of Dop1R1 (up) and Dop2R (down) in the MB lobes. Dop1R1; γ lobe: *P* = 0.9226, β lobe: *P* > 0.9999, β′ lobe: *P* > 0.9999, α lobe: *P* = 0.0479, α′ lobe: *P* = 0.1819 (Sidak’s multiple comparison). *n* = 10 for each group. Dop2R; γ lobe: *P* = 0.9899, β lobe: *P* > 0.9999, β′ lobe: *P* > 0.9999, α lobe: *P* = 0.9999, α′ lobe: *P* = 0.8845 (Sidak’s multiple comparison). *n* = 5 for each group. (**e**) Genetic overexpression of *Dop1R1* enhances ethanol preference. *Dop1R1* is overexpressed using the *Dop1R1* knock-in *2A-GAL4* driver and 15% ethanol preference is measured for one day. *F*_(2, 41)_ = 5.712, *P* = 0.0065 (one-way ANOVA), *Dop1R1-GAL4/w* vs. *Dop1R1-GAL4/UAS-Dop1R1*: *P* = 0.0302, *Dop1R1-GAL4/UAS-Dop1R1 vs. w/UAS-Dop1R1*, *P* = 0.0055, *n* = 14, 16, 14 for *Dop1R1-GAL4/w*, *Dop1R1-GAL4/UAS-Dop1R1* and *w/UAS-Dop1R1*, respectively (Dunnett’s multiple comparison). (**f**) Model. Repeated ethanol intake alters the dopamine synapses by elevating the protein level of Dop1R1, thereby promotes further intake. DAN: dopamine neurons. KC: Kenyon cells. Bar graphs: mean ± SEM. *: *P* < 0.05.

The selective requirement of Dop1R1 for acquired, but not acute, ethanol preference prompted us to hypothesize that voluntary intake of ethanol might change the Dop1R1 signaling. To test this hypothesis, we quantified the protein level of Dop1R1 and Dop2R, which mediate the opposite effects^51^, in the brain using the Venus tagged endogenous dopamine receptors^52^. Strikingly, when the flies were given an access to the ethanol-containing food for 3 days, the protein level of Dop1R1, but not Dop2R, was significantly increased in the medial lobe of the MB (Fig. 5a,b). In contrast, addition of methamphetamine did not lead to the Dop1R1 augmentation (Fig. 5c,d). We then hypothesized that the increased Dop1R1 level is sufficient to enhance ethanol preference. Indeed, we found that genetic overexpression of *Dop1R1* enhanced ethanol preference (Fig. 5e). Therefore, these results suggest that voluntary ethanol intake increases protein level of Dop1R1, which further mediates the acquired ethanol preference (Fig. 5f).

## Discussion

In this study, we systematically tested the preference and its experience-dependent changes of five different drugs using a two-choice feeding assay (Fig. 1). Flies showed a robust preference to ethanol, which escalated over time. A previous study has shown that it occurs in a wide range of the ethanol concentration^12^. In contrast, they avoided cocaine, caffeine and morphine, all of which are plant alkaloid. They even developed an aversion to morphine (Fig. 1b). This diversity might be explained by their evolutionally background: many of the *Drosophila* species are naturally feeding on fruits, and *melanogaster* particularly prefers fermented rotten fruits^53^, which is the major source of ethanol in the wild^54^. Therefore, ethanol might function as an indicator of a good food resource, which urges them to repeatedly consume (Fig. 2). Plant alkaloids on the other hand are mainly defensive compounds against herbivore insects^55^. Indeed, cocaine and caffeine prevent the larvae of *Manduca sexta* from eating plant leaves and eventually kill them^56,57^. Also for flies, cocaine shortens the lifespan and disrupts oogenesis^58^, and caffeine prevents food intake^59^. Our result revealed that *Drosophila* can readily avoid these plant alkaloids, supporting an idea that drug intake behavior is shaped by the adaptive evolution^60,61^.

Methamphetamine is an artificial compound and was preferred at the beginning of the measurement (Figs. 1, 3). Analysis of the mutants and the genetic knock-down suggests that the preference is dependent on Dop1R1 and Dop1R2, reminiscent of sugar-rewarded appetitive memory^33,62,63^ (Fig. 4d). A recent genome wide association study consistently identified the *Dop1R1* gene as a critical regulator of consumption of methamphetamine^64^. Interestingly for ethanol, the *Dop1R1* mutant showed normal or slightly enhanced preference on the first day of the measurement but no significant increase on the following days (Fig. 4a). These observations clearly indicate the involvement of the dopamine system, but with different temporal dynamics among these two drugs. Because methamphetamine directly induces a robust dopamine release, primarily by induction of reverse transport through monoamine transporters^21,46,65^, it may be strong enough to stimulate Dop1R1 to promote the drug intake from the beginning. Ethanol on the other hand does not strongly activate the rewarding dopamine neurons at the first exposure but the response develops afterwards^38^. We found that self-administration of ethanol, but not methamphetamine, enhanced the Dop1R1 protein level (Fig. 5). These lines of evidence support an idea that repeated ethanol intake, but not methamphetamine, sensitizes the dopamine-Dop1R1 signaling, possibly by increasing both the dopamine release and the receptor. The sensitized reward signaling might escalate self-administration of ethanol (Fig. 5f). A recently reported switch of Dop2R isoforms in the MB after repeated ethanol exposure^40^ might also contribute to the behavioral change. It would be therefore important in the future to investigate at which level, i.e. transcription, translation or protein turnover, the experience-dependent changes take place and how they are regulated.

*DopEcR* mutant on the other hand showed a higher preference for both ethanol and methamphetamine (Fig. 4a,d). The *DopEcR* mutant flies are less sensitive to ethanol sedation^66,67^, and show defects in dealing with various stressors such as starvation, heat and sexual rejection^68–70^. Therefore, DopEcR might mediate aversive effects of drugs, which include ethanol sedation, thereby function as a ‘brake’ for the further drug intake. Consistently, a prospective clinical study in humans has shown that lower sensitivity to ethanol sedation predicted a greater number of alcohol use disorders afterwards^71^. Closer look at the interaction of the accel and the brake would help understanding the neural mechanisms of the drug intake behavior.

## Methods

### Flies

Flies were reared in a mass culture on standard cornmeal food at 24 °C under 12–12 h light–dark cycles. *Canton-S* was used as the wild-type. Following dopamine receptor mutants were used: *dumb*^*2*^^48^, *Dop1R2*^*attP*^^72^, *Dop2R*^*∆1*^^73^ and *DopEcR*^*GAL4*^^66^. Following transgenic strains were used: *w*^*1118*^*;;R58E02-GAL4*^33^ (BDSC #41347), *w*^*1118*^*;;UAS-Shibirets1* (pJFRC100)^74^, *w*^*1118*^*;MB010B-GAL4*^49^ (BDSC #68293), *y*^*1*^*v*^*1*^*;UAS-Dop1R1.RNAi* (P{TRiP.HMC02344})^75^ (BDSC #55239), *w*^*1118*^*;;Dop1R1-GAL4*^52^*, y*^*1*^*w*^*1118*^*;;Dop1R1-Venus*^52^, *y*^*1*^*w*^*1118*^*,Dop2R-Venus*^52^ and *w*^*1118*^*,UAS-Dop1R1*^76^.

### CAFE assay

CAFE assay was performed referring to the original study^44^ with some modifications. Male and female flies were separated within 1 day after eclosion and 4–7 days old flies were used for the measurement. A group of four male flies (Figs. 1, 4, 5) or a single male fly (Figs. 2, 3) was placed in a column-shaped plastic container, to which two capillaries were inserted. Female flies were used for the shibire^ts1^ blockade (Fig. 4c), because of the high mortality when male flies were measured at the high temperature. Capillaries with inner diameter of 0.5 mm (BF100-50–15, Sutter Instrument, CA, USA) or 0.021 mm (BR708707, BRAND GMBH, Germany) were used for grouped or single flies, respectively. Sucrose (S9378, Sigma-Aldrich, St. Louis, MO, USA), ethanol (09-0851-5, Sigma-Aldrich), caffeine (C8960, Sigma-Aldrich), cocaine hydrochloride (Takeda Pharmaceutical, Tokyo, Japan), methamphetamine hydrochloride (Sumitomo Dainippon Pharma, Osaka, Japan) and morphine hydrochloride (Daiichi-Sankyo, Tokyo, Japan) were dissolved into the evian mineral water (Danon, Paris, France) with indicated concentrations. The sucrose concentration (5%) was chosen following previous studies that measured ethanol preference^12,41,43,44^. Sulforhodamine B sodium salt (S1402, Sigma-Aldrich) was added by 0.005% (w/v, Figs. 1, 4, 5) or 0.02% (Figs. 2, 3) to stain the solution. n-Octyl Acetate (0.001%, A6042, Toyo Chemical Industry, Tokyo, Japan) and vanillin (0.0001%, H0264, Tokyo Chemical Industry) were reciprocally added to the two capillaries to give an odor cue for the flies: half of the groups were presented with the drugs flavored with n-Octyl Acetate and the other half with vanillin. Experiments were performed in a plexiglas box containing wet tissue paper to minimize evaporation in 12 h-12 h light–dark cycles. Temperature was set to 24 °C, except for the shibire^ts1^ blockade experiment (Fig. 4c), which was performed at 30 °C. Capillaries were pictured every day (Figs. 1, 4, 5) or every 5 min (Figs. 2, 3) using the Pentax Q-S1 camera (Ricoh, Tokyo, Japan). Evaporation was measured for each drug and dose in parallel by imaging the capillaries without flies.

Daily consumption was calculated as descent of liquid level subtracted by evaporation, divided by the number of living flies. Preference index was calculated as

$$\frac{\text{Consumption of the drug solution }-\text{ Consumption of the control solution}}{\text{Total consumption}}$$

To detect each meal event (Figs. 2, 3), a threshold was calculated for each time bin as mean evaporation + (standard deviation of evaporation) * 2 using measurements of evaporation without flies. A descent of liquid level bigger than the threshold was defined as a meal bout.

### Immunohistochemistry

Male flies harboring the Venus-tagged dopamine receptors^52^ were presented with 5% sucrose solution (“Sugar only”) or 5% sucrose solution and 5% sucrose solution supplemented with 15% ethanol (“With ethanol”) in the CAFE assay chamber for 3 days. Fly brains were then dissected in PBS, immediately fixed in 2% paraformaldehyde in PBS for 1 h at room temperature, and washed three times with PBST (0.1% Triton X-100 in PBS). The brains were then blocked with 3% goat serum in PBST for 30 min at room temperature and incubated in the primary antibody solution (rabbit anti-GFP (1:1000; catalog #A11122, Thermo Fisher Scientific, MA, USA) and 1% goat serum in PBST) and in the secondary antibody solution (Alexa Fluor 488 goat antirabbit (1:1000; catalog #A11034, Thermo Fisher Scientific) and 1% goat serum in PBST) at 4 °C for two nights respectively. Brains were then washed for three times with PBST and mounted in SeeDB2G^77^. Images were obtained using the Olympus FV1200 confocal microscope (Olympus, Tokyo, Japan) with the 20x, 0.85NA oil objective lens (UPLSAPO20XO, Olympus). Images of the two groups were acquired at the same time periods under the identical microscope settings.

### Statistics

Statistical tests were performed on GraphPad Prism 8 for macOS (GraphPad Software, CA, USA). One-sample *t*-test, repeated measures one-way analysis of variance (ANOVA) or ordinary one-way ANOVA followed by Dunnett’s or Sidak’s multiple comparison was performed when the assumption of normal distribution (D'Agostino & Pearson test) was not violated. Two-stage linear step-up procedure of Benjamini, Krieger and Yekutieli was performed to compare the consumption of the wild type flies (Fig. 1c). Otherwise nonparametric statistics, i.e. Wilcoxon test, Friedman test or Kruskal–Wallis test followed by Dunn’s multiple comparison was performed. The significance level of statistical tests was set to 0.05.

## Supplementary Information

Supplementary Information.

## Data Availability

The datasets generated during the current study are available from the corresponding author T.I. on reasonable request.
