# Supplementary material for: Voluntary intake of psychoactive substances is regulated by the dopamine receptor Dop1R1 in Drosophila
Source: Sci Rep. 2021 Feb 9;11:3432. doi: 10.1038/s41598-021-82813-0 (PMC7873259; doi:10.1038/s41598-021-82813-0)
Supplement: Supplementary file 1 — Supplementary Information. [file 41598_2021_82813_MOESM1_ESM.pdf]

Supplementary information for

Voluntary intake of psychoactive substances is regulated by the dopamine receptor Dop1R1 in *Drosophila*

Authors: Mai Kanno [1], Shun Hiramatsu [1], Shu Kondo [2], Hiromu Tanimoto [1], Toshiharu Ichinose [1,3,4,5,*]

1. Graduate School of Life Sciences, Tohoku University, Sendai, 980-8577, Japan

2. Invertebrate Genetics Laboratory, National Institute of Genetics, Mishima, 411-8540, Japan

3. Frontier Research Institute for Interdisciplinary Sciences, Tohoku University, Sendai, 980-8578, Japan

4. Center for Transdisciplinary Research, Niigata University, Niigata, 950-2181, Japan

5. Department of Neuropharmacology, Nagoya City University, Nagoya, 467-8603, Japan

***Correspondence to:**

Toshiharu Ichinose PhD

Frontier Research Institute for Interdisciplinary Sciences, Tohoku University

Email: toshiharu.ichinose.c1@tohoku.ac.jp

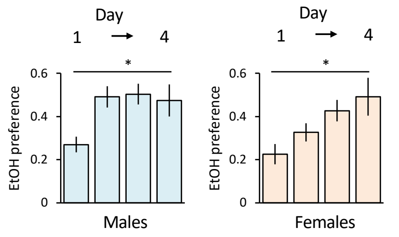

Supplementary figure 1. Both males and females acquire preference for ethanol. Groups of four wild-type male (left) or female (right) flies are given a choice between 5% sucrose and 5% sucrose supplemented with 15% ethanol, similar to figure 1, and daily preference is plotted. Male: $P = 0.0075$, $n = 15$ (Friedman test); female: $F_{(1.939, 27.14)} = 5.174$, $P = 0.0155$, $n = 15$ (one-way ANOVA).
